# Do Different Approaches Make a Difference in Isokinetic Performance in Elderly Patients With Femoral Neck Fracture Who Underwent Bipolar Endoprosthesis?

**DOI:** 10.7759/cureus.33362

**Published:** 2023-01-04

**Authors:** İzzet Korkmaz, Nurdan Korkmaz, Saadet S Koç, Olgun Bingöl, Enver Kılıç, Guzelali Ozdemir, Güray Toğral

**Affiliations:** 1 Orthopedics and Traumatology, Ankara City Hospital, Ankara, TUR; 2 Physical Medicine and Rehabilitation, Gaziler Physical Medicine and Rehabilitation Training and Research Hospital, Ankara, TUR; 3 Physical Medicine and Rehabilitation, Ankara City Hospital, Ankara, TUR; 4 Orthopedics and Traumatology, Dr. Abdurrahman Yurtarslan Oncology Training and Research Hospital, Ankara, TUR

**Keywords:** approach, isokinetic performance, femoral neck fracture, bipolar hemiarthroplasty, hip

## Abstract

Background

There are ongoing doubts about the effects of the commonly used anterolateral approach (ALA) and posterolateral approach (PLA) for bipolar hemiarthroplasty (BHA) on hip muscle strength after surgery. In this study, it was aimed to evaluate the isokinetic performance of the operated and non-operated hips in patients with femoral neck fractures who underwent BHA with PLA or ALA and to compare the isokinetic performance of the hips and functional results between the two approaches.

Materials and methods

Forty-one patients who underwent unilateral BHA with PLA or ALA for femoral neck fracture between February 2019 and December 2020 were enrolled. The isokinetic performance of the flexor, extensor, and abductor muscles of the operated and non-operated hips were evaluated by measuring peak torque, total work, and average power. Functional status was assessed using Harris Hip Score and Short Form 36.

Results

The patients were divided into two groups; those operated with PLA (n=22) and with ALA (n=19). The groups had similar demographic and clinical characteristics. All isokinetic parameters of the operated hips did not differ between the groups (all p>0.05). In both groups, all isokinetic parameters were significantly lower in the operated hips than in the non-operated hips.

Conclusion

Although there are debates about potential extensor muscle injury with PLA and potential abductor muscle injury with ALA, this study showed that functional results and the isokinetic performance of both approaches were not different.

## Introduction

Femoral neck fractures are common in elderly people. Female gender, low bone mineral density, and limitation of movement are among the most common causes that increase the risk of femoral neck fracture [1,2]. Early mobilization and minimizing complications increase the success of the treatment in displaced femoral neck fractures, so the choice of surgical approach is important [3].

Bipolar hemiarthroplasty (BHA) is a common surgical procedure used to treat displaced fractures of the femoral neck in the elderly [4]. The posterolateral approach (PLA) and the anterolateral approach (ALA) is the commonly used approaches, according to the national database that collects data on surgical approaches used for BHA [5-7]. The advantages of PLA are that it protects the abductor mechanism, minimizes intraoperative bleeding, and reduces the risk of heterotopic ossification compared to ALA [8-10]. Its main disadvantage is that it increases the risk of injury to the hip external rotators and potential hip dislocation. It has been reported that the rate of hip dislocation is higher in PLA than in ALA [11,12]. In ALA, the external rotator of the hip is preserved while the abductor mechanism is damaged. Damage to the abductor mechanism causes the contralateral pelvis to drop during a single-leg stance and clinically manifests as Trendelenburg gait [13]. Loss of abduction muscle strength may impair balance and increase the risk of falling, especially in the elderly population [14].

Therefore, the surgical approach used in BHA may be important for operative success and recovery of function. However, current guidelines on the surgical approach are based on limited evidence. This situation causes surgeons to choose an approach according to their education and experience [4,7]. We think that it is clinically important to evaluate hip muscle strength with objective measurement methods in patients operated on with these approaches. However, to the best of our knowledge, there is no study that measures the effect of these two approaches on hip muscle strength in femoral neck fractures by isokinetic assessment. Therefore, this study aimed to evaluate the isokinetic performance of the operated and non-operated hips in the patients who underwent BHA with PLA or ALA and to compare the isokinetic performance of the hips and functional results between the two approaches.

## Materials and methods

Patients and ethics statement

Patients who applied to the orthopedics and traumatology clinic of a regional trauma center between February 2019 and December 2020 and underwent BHA for femoral neck fracture were evaluated retrospectively. Institutional Ethics Committee approval was obtained (E1-20-1095). All procedures of the study were carried out in accordance with the Declaration of Helsinki. The patients included in the study were informed about the entire study before the isokinetic procedures, and their written consent was obtained.

Male and female patients with a femoral neck fracture, aged 65-90 years, undergoing BHA with PLA or ALA, and who were present for the 12-month follow-up after surgery were included in the study. Patients with a subtrochanteric femur fracture, pathological fracture, hip revision surgery, multiple fractures, isolated fractures of the greater and lesser trochanter, malignancy, surgery with an approach other than PLA or ALA, and no follow-up were excluded from the study. All patients had been operated on with spinal anesthesia and cementless BHA in a single center.

The sample size estimation was made using the G*Power software (version 3.1.9.4). Based on the study of Bertocci et al. [15], flexion peak torque per body weight was taken as the primary outcome measure. In the analysis performed by taking the values of 12.5 ± 5.4 in the operated hip and 20.0 ± 11.3 in the healthy hip, the minimum number of patients was found to be at least 18 for each group with 80% power and 5% probability of type I error.

**Figure 1:**
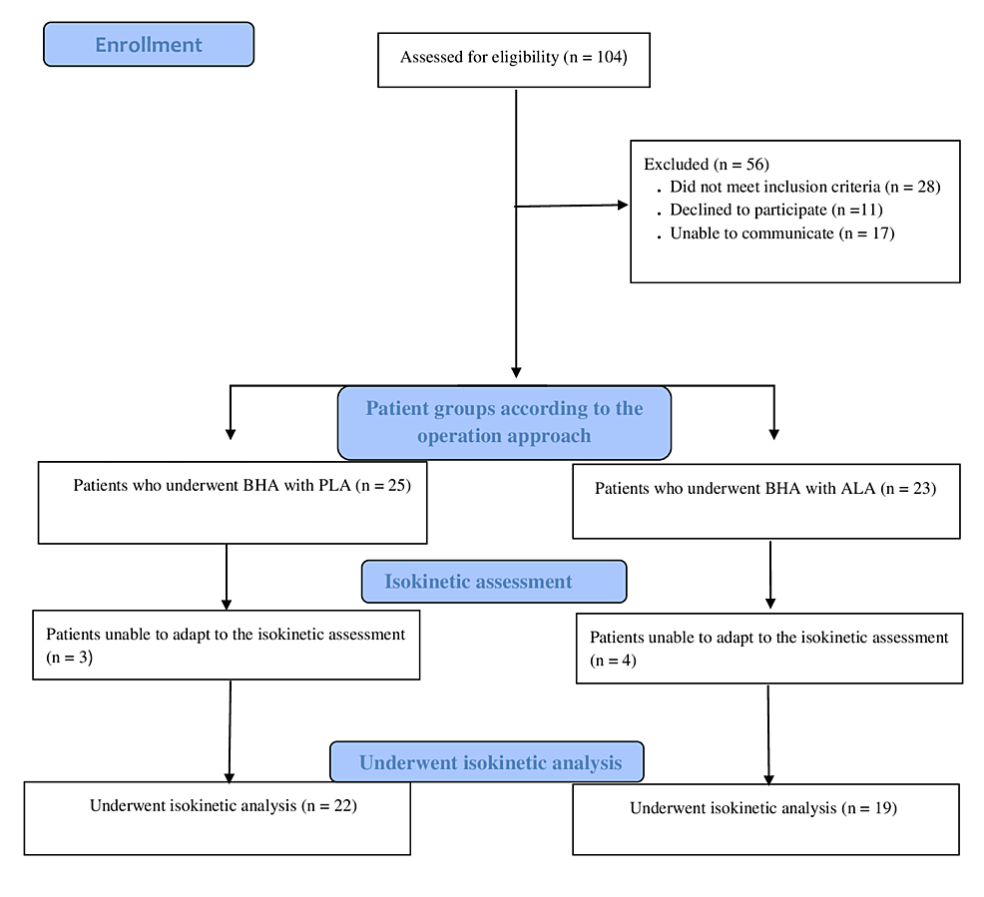
Consort diagram of the patients PLA - posterolateral approach; ALA - anterolateral approach

One hundred four patients who underwent BHA for femoral neck fracture between February 2019 and December 2020 were identified. Twenty-eight patients did not meet the inclusion criteria, 11 did not want to participate in the study, 17 could not be reached, and so a total of 56 patients were excluded from the study. Of the 48 patients included in the study, 25 were patients who applied BHA with PLA and 23 with ALA. Three of the patients who underwent BHA with PLA and four of the patients who underwent BHA with ALA could not adapt to the isokinetic assessment. As a result, 22 patients who underwent BHA with PLA and 19 patients who underwent BHA with ALA were analyzed isokinetically (Figure 1).

Age, gender, height, weight, operated side, surgical approach, and anesthesia type were recorded. The functional status of all patients before isokinetic performance measurement was assessed using Short Form 36 (SF-36) and Harris Hip Scores (HHS).

Surgical techniques

BHA was applied to patients with either a PLA or ALA. In PLA, a Kocher-Langenbeck incision was made with the fractured hip joint up, in the lateral decubitus position, and the knee joint flexed to at least 45 degrees. After passing the layers with appropriate dissection, the short external rotators, except for the quadratus femoris, were separated from the greater trochanter and suspended. The hip capsule was opened posteriorly in a T-shape parallel to the femoral neck. In the final stage, the posterior capsule was repaired, and the external rotators were sutured to the anatomical location [16]. ALA was first identified by Sir Watson Jones in 1936 and is still used in hip arthroplasty operations. In this approach, the space between the gluteus medius and tensor fascia lata was used with the patient in the supine position. The muscles were separated by appropriate dissection, and the joint capsule was opened parallel to the femoral neck. In the final stage, all layers were repaired anatomically. The advantages of this approach include correct positioning of the acetabular cup, a very low rate of dislocation, low vascular or neurological risks, and a low rate of deep venous thrombosis [17].

A single dose of first-generation cephalosporin was administered to all patients before the surgery. Low molecular weight heparin was injected subcutaneously for three weeks, and anti-embolic stockings were worn for deep vein thrombosis prophylaxis after the surgery. In addition, all patients did not receive any physical therapy and rehabilitation programs other than activities of daily living and mobility training during their hospital stay.

Isokinetic performance measurements

**Figure 2:**
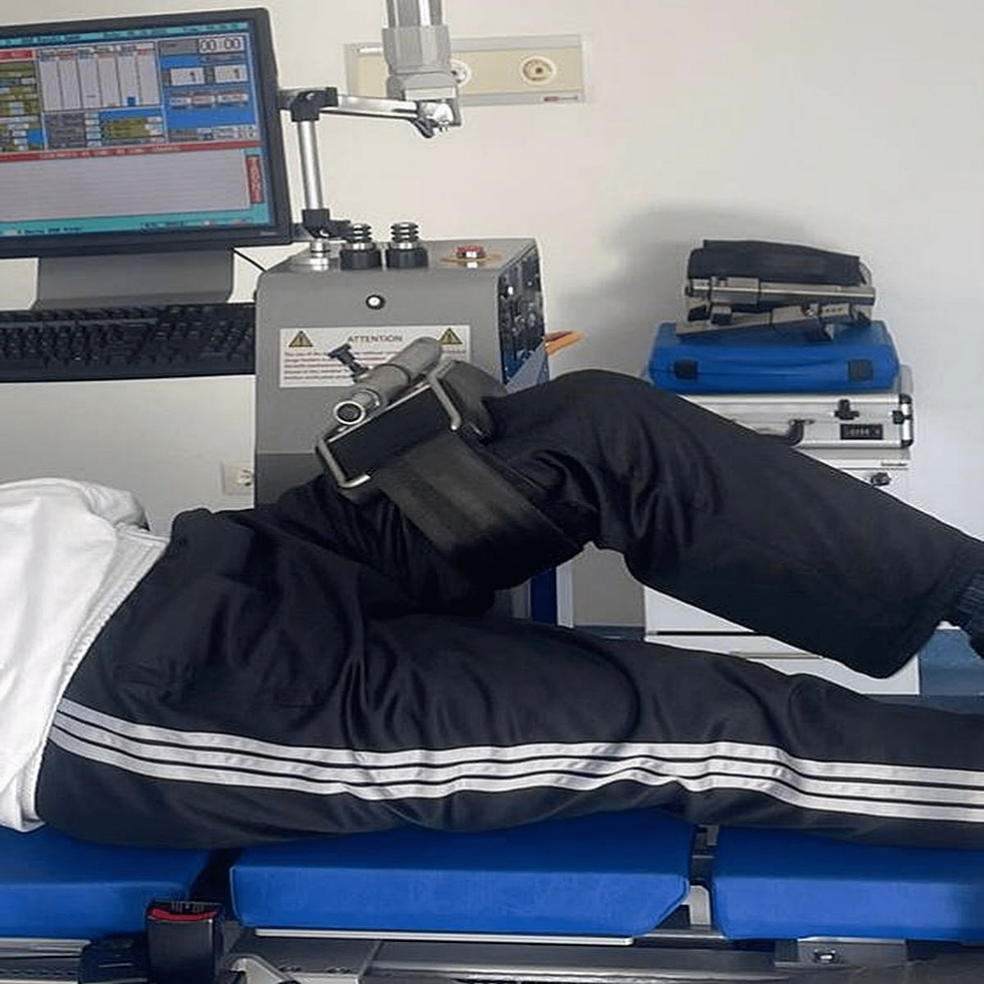
Isokinetic measurement of hip flexion and extension in the supine position

**Figure 3:**
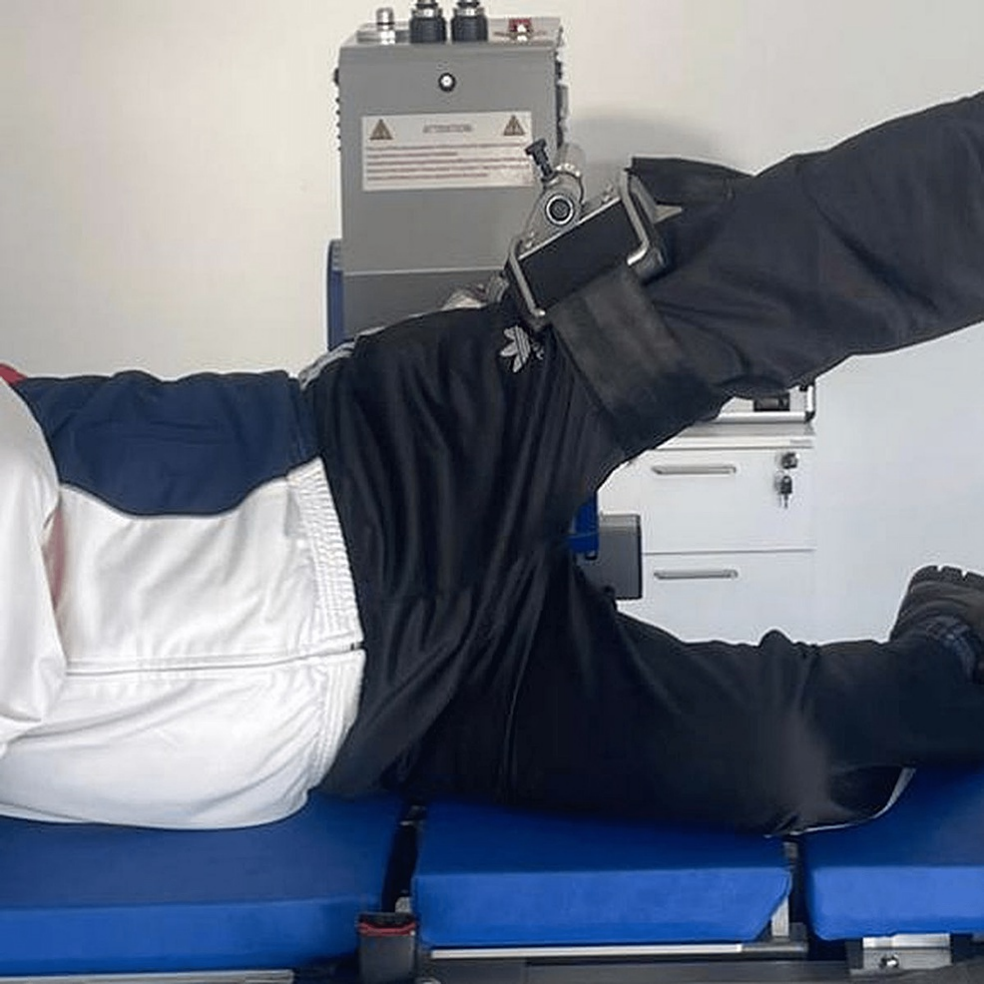
Isokinetic measurement of hip abductor muscles in the lateral decubitus position

Isokinetic measurements were performed with the ISOMED 2000 E750 (D. & R. Ferstl GmbH, Hemau, Germany) device in the presence of a single physiatrist and physical therapist blinded to the study groups. The isokinetic values were measured as peak torque (PT), total work (TW), and average power (AP) of the flexion, extension, and abduction muscles in the non-operated and operated hips. All measurements were assessed at the postoperative 12-month follow-up. TW was expressed in joule (J), PT in Newton-meters (Nm), and AP in watts (W). In order to evaluate flexion and extension measurements, the procedure was performed in a supine position with the hip to be tested close to the dynamometer (Figure 2), and to evaluate abduction measurements, the procedure was performed in a lateral decubitus position with the hip to be tested on top (Figure 3). The chair and dynamometer shaft were adjusted to align with the hip rotation axis. The axis of rotation of the hip joint was adjusted at the level of the greater trochanter when assessing the hip flexors and extensors and at the anterior superior iliac level when assessing the hip abductors. Then gravity correction was made [18], and the mode of concentric muscle contraction was selected. Isokinetic hip flexion, extension, and abduction were evaluated at a preset velocity of 60°/sec in the widest range of motion the patient could make. All these steps were performed for both the operated and non-operated hips.

Statistical analysis

Statistical analyses were performed using the SPSS version 22.0 software program for Windows (IBM Inc., Armonk, New York). Descriptive data were expressed as percentages and frequencies for categorical variables and as mean, standard deviation, median, and minimum-maximum values for numerical variables. In the normality analysis, non-parametric test procedures were used because the numerical values did not show the normal distribution in the Shapiro-Wilk test. In this context, Mann-Whitney U Test and Wilcoxon signed-rank test were used to determine the relationships between parameters. The Chi-square test was used in the analysis of categorical variables. The results were evaluated within the 95% confidence interval, and a p<0.05 value was considered significant.

## Results

Forty-one patients who were operated on within the specified time period, met the study criteria and were accepted to participate in the study, were divided into two groups, ALA (n=19) and PLA (n=22), according to the applied surgical approach. There was no statistically significant difference in the parameters of age, gender, operation side, body mass index, and length of hospital stay between both groups (Table 1).

**Table 1:** Demographic and clinical characteristics of the patients PLA -posterolateral approach; ALA - anterolateral approach; BMI - body mass index

	PLA (n=22)	ALA (n=19)	p-value
Age (years)	73.32 ± 6.74	75.79 ± 7.79	0.372
Gender, male / female	8 / 14	7 / 12	0.975
BMI	27.54 ± 3.95	26.24 ± 4.97	0.340
Operated side, right / left	10 / 12	9 / 10	0.902
Length of hospital stay (days)	4.50 ± 0.67	4.58 ± 0.51	0.868

When the isokinetic performances of the operated and non-operated hips were compared within the groups, all examined isokinetic parameters (PT, TW, AP) showed a significant difference, and these parameters were significantly reduced in the operated hips compared to the non-operated hips (all p<0.05). In addition, operated hips and non-operated hips were compared between the groups themselves, but there was no significant difference between the isokinetic parameters of both operated and non-operated hips (all p>0.05) (Table 2).

**Table 2:** Comparison of isokinetic parameters of bipolar hemiarthroplasty patients' operated and non-operated hips within and between groups PLA - posterolateral approach; ALA - anterolateral approach The values were presented as mean ± standard deviation * - statistically significant

Variable	Hip joint	PLA (n=22)	ALA (n=19)	p-value
Flexion peak tork	Operated	13.77 ± 11.24	16.95 ± 15.84	0.627
	Non-operated	25.77 ± 13.66	23.79 ± 19.59	0.326
	p-value	0.001*	0.001*	
Extension peak tork	Operated	11.59 ± 14.20	18.21 ± 17.55	0.146
	Non-operated	20.41 ± 15.23	23.37 ± 22.64	0.990
	p-value	0.001*	0.007*	
Abduction peak tork	Operated	9.36 ± 9.69	12.53 ± 11.70	0.409
	Non-operated	18.09 ± 13.00	19.68 ± 15.07	0.824
	p-value	0.005*	0.001*	
Flexion total work	Operated	31.32 ± 53.49	68.11 ± 117.55	0.546
	Non-operated	48.68 ± 72.24	102.21 ± 165.19	0.844
	p-value	0.001*	0.001*	
Extension total work	Operated	26.00 ± 51.81	78.16 ± 132.04	0.101
	Non-operated	42.86 ± 73.26	103.58 ± 166.83	0.666
	p-value	0.001*	0.001*	
Abduction total work	Operated	9.41 ± 26.62	29.16 ± 49.35	0.228
	Non-operated	15.00 ± 29.26	56.26 ± 83.38	0.926
	p-value	0.004*	0.007*	
Flexion average power	Operated	5.05 ± 4.36	7.84 ± 7.91	0.378
	Non-operated	10.55 ± 4.63	10.79 ± 8.42	0.436
	p-value	0.001*	0.001*	
Extension average power	Operated	3.73 ± 3.51	6.32 ± 5.12	0.116
	Non-operated	7.05 ± 4.79	9.58 ± 8.50	0.833
	p-value	0.001*	0.001*	
Abduction average power	Operated	2.05 ± 3.00	3.68 ± 4.37	0.221
	Non-operated	4.45 ± 4.34	5.58 ± 5.82	0.661
	p-value	0.001*	0.011*	

Postoperative SF-36 and HHS values ​​of both groups did not differ significantly (all p>0.05) (Table 3).

**Table 3:** Functional status parameters of the groups 12 months after the operation PLA - posterolateral approach; ALA - anterolateral approach The values were presented as mean ± standard deviation

	PLA (n=22)	ALA (n=19)	p-value
Short Form 36	95.45 ± 19.36	98.53 ± 3.81	0.518
Harris Hip Score	79.64 ± 9.92	77.11 ± 12.08	0.388

## Discussion

This study examined the hip isokinetic performance of patients who applied BHA with PLA or ALA. To the best of our knowledge, hip isokinetic performance measurements have been studied mostly in patients who underwent total hip arthroplasty, and this study is the first in patients who underwent BHA. The results of the study demonstrated that there was a significant decrease in flexion, extension, and abduction isokinetic performances of the operated hips compared to the non-operated hips one year after the operation, regardless of which approach was operated. All isokinetic performance results were found to be similar in hips applied BHA with PLA or ALA. In addition, the postoperative functional status reported by the patients did not differ between the two groups. No complications were observed in all patients.

In the literature, there are concerns regarding potential abductor muscle damage during ALA [14]. However, Cankaya et al. obtained similar isokinetic results with PLA at six and 12 months in patients who underwent total hip arthroplasty in their randomized controlled trial [19]. In two prospective studies comparing the anterior approach and direct lateral approach with PLA, it was revealed that abductor strength was significantly lower in the postoperative 12th month in patients operated with the anterior and direct lateral approach [20,21]. It has also been suggested that damage to the hip extensor muscle group may occur with the PLA approach [11,12]. However, Imren et al. found that there was no significant decrease in hip extension strength in patients who underwent total hip arthroplasty with PLA [22]. In this study, the isokinetic values ​​of both the operated and non-operated hips did not show a statistically significant difference between the PLA and ALA groups. In other words, no difference was found between the two groups in all isokinetic parameters, including extensor muscle strengths that could potentially be affected in the PLA group and abductor muscle strength in the ALA group in the operated hips. These results suggested that the loss of strength in the operated hips is independent of the surgical approach.

In a study, no statistically significant difference was found between the isokinetic hip muscle strengths between the dominant and non-dominant sides [23]. Several studies have compared the isokinetic strength of the operated and non-operated hips of patients undergoing total hip arthroplasty. Bertocci et al. found no significant difference in terms of isokinetic performance between operated and non-operated hips in patients who underwent total hip arthroplasty, and they attributed this to the fact that all patients received effective physical therapy after the surgery [15]. In addition, another study in which patients received an effective physical therapy and rehabilitation program after the operation, and the mean age was 62 years, did not find a significant difference between operated and non-operated hips [24]. In the study of Long et al., the operated and non-operated hips of patients with total hip arthroplasty, whose mean age was 55, were compared, and strength losses were found to be 8-14% in the first year and 5-24% in the second year, but this was not statistically significant [25]. However, Borja et al. showed significant differences between the isokinetic performance of the operated and non-operated hips in patients who underwent total hip arthroplasty with the posterior approach [26]. In this study, the isokinetic performance of the non-operated hips was used to represent healthy control subjects [23]. Flexion, extension, and abduction isokinetic performance of the operated and non-operated hips were statistically different, and there was a significant loss of strength in all directions in the operated hips. We think that this may be due to the fact that the patients in this study were older than previous studies and did not receive physical therapy and rehabilitation after the operation.

Due to the risk of postoperative dislocation in BHA surgeries, it has been recommended to leave the PLA [27]. The dislocation was attributed to the fact that the posterior capsule remained intact in the anterior approach but was damaged in the PLA [28]. No dislocations occurred in the operated hips in this study. We attribute this to the fact that the capsule was repaired and the external rotators attached to the anatomical localization at the end of the surgery.

The main limitation of this study is to compare and discuss the results of the study with the results of the total hip replacement study since there is no similar study performed with BHA operation. Despite the limitations, the superiority of the study is that it is the first to evaluate isokinetic hip performance in elderly patients who underwent BHA for femoral neck fracture.

## Conclusions

Although there are debates about potential extensor muscle injury during PLA and potential abductor muscle injury during ALA, this study showed that functional results and the isokinetic performance of both approaches were not different. However, it was determined that there may be some loss in the isokinetic performance of flexion, extension, and abduction of the operated hips compared to the non-operative hips. These suggested that the loss of strength in the operated hips was not related to the surgical approach.
